# Deliberately Retrieved Negative Memories Can Improve Mood Beyond the Intention to Do So

**DOI:** 10.5964/ejop.4629

**Published:** 2022-08-31

**Authors:** Veronika V. Nourkova, Alena A. Gofman

**Affiliations:** 1 Department of General Psychology, Lomonosov Moscow State University, Moscow, Russian Federation; Department of Education, Psychology, Communication, University of Bari ‘Aldo Moro’, Bari, Italy

**Keywords:** emotion regulation, autobiographical memory function, reappraisal, distraction, downward comparison, vicarious memories

## Abstract

The role of autobiographical memory in emotion regulation is deemed as limited to the selective retrieval of positive memories intended as a distraction from unpleasant stimuli. The present experimental study is the first to examine whether negative autobiographical memories serve as a way to boost one’s mood by employing the mechanism of retrospective downward autobiographical comparison between now and then. We hypothesised that this mechanism may operate in response to negative memories, leading to positive mood induction. Ninety-nine students participated in four memory tasks: autobiographical positive, autobiographical negative, vicarious positive, and vicarious negative. Emotional states at pre- and post-tests were assessed using the implicit test differentiating positive (PA) and negative (NA) components of mood. The results replicated previous studies on the mood-repair effect of deliberate positive recall. The most striking finding is that negative autobiographical recall consistently boosted PA and inhibited NA. This result supported the idea of retrospective downward autobiographical comparison as a plausible mechanism behind the efficacy of negative memories in emotion regulation.

Folk theory and scientific psychology converge on the idea that healthy, non-depressed individuals can repair their negative moods by retrieving positive personal memories. Popular Internet resources advise unearthing and browsing sweet memories from childhood, vacations, and family reunions to overcome melancholy. When psychologists in a lab asked naïve participants whether they agree with the statement ‘When a person is feeling bad, it is helpful to recall pleasant memories’, they typically marked 4 or 5 on a 5-point Likert scale, endorsing the use of positive memories to regulate emotions (Wang, Koh, Song, & Hou, 2015). Experimental psychologists and clinicians suggest that difficulties in employing positive memories in mood regulation play an important role in various emotional disorders, such as depression and anxiety (McKay, Singer, & Conway, 2012; Singer, 2006).

## Memory in Emotion Regulation

Harris, Rasmussen, and Berntsen (2014) justified the proposition that emotion regulation is a distinctive function of autobiographical memory in the exhaustive literature review. For instance, several studies have indicated that individuals deliberately recruit positive memories to resist a natural mood-congruent recall, followed by negative mood induction (Joormann, Siemer, & Gotlib, 2007; Josephson, Singer, & Salovey, 1996; McFarland & Buehler, 1997).

Similarly, Öner and Gülgöz (2018) asked participants to recall three memories that made them feel sad or angry and then asked them to retrieve another memory with no specific instruction of emotion. Their results demonstrated two things. First, participants were able to induce negative affect by retrieving appropriate negative memories. Second, they successfully repaired their emotional states through subsequent positive recall. Furthermore, if the regulatory goal is to enhance positive affect, people deliberately select happier autobiographical memories in comparison to accomplishing the goals of self-continuity, social bonding, and directing behaviour (Wolf & Demiray, 2019).

The previous literature has consistently shown that the most common outcome of autobiographical memory dynamics over time is a positive bias, which includes the fact that good memories persist longer than bad memories and, in general, makes memories more pleasant than experiences behind those memories. This positive bias is thought to assist in maintaining a hopeful outlook about the future and providing a means for situational emotion regulation in an automatic manner without purposive intention to do so (Walker & Skowronski, 2009). Therefore, reminiscing may be a powerful emotion regulation resource. It raises a question regarding the underlying mechanisms.

Although several frameworks conceptualising the different ways in which people regulate their emotions have been proposed and empirically tested (see for review, Webb, Miles, & Sheeran, 2012), there are two strategies that incorporate memories. These strategies derived from the process model described by Gross (2015) and include attentional deployment in the form of distraction and cognitive change in the form of reappraisal.

Regarding the mnemonic impact on emotion regulation, distraction ‘moves attention away from the situation altogether’ and ‘may also involve changing internal focus, such as when individuals invoke thoughts or memories that are inconsistent with the undesirable emotional state’ (Gross & Thompson, 2007, p. 13). It is a trivial assumption that positive autobiographical memories are very probable candidates for positive distraction, since recall reinstates the emotions associated with the addressed experiences. It was shown that after negative mood induction, non-depressed adults equally benefited from the instructed retrieval of positive memories and attention re-focusing (Joormann & Siemer, 2004). Congruently, Kovacs et al. (2015) found similar mood boosts after both distraction (in the form of watching shapes via kaleidoscope) and recall of two consecutive happy memories in never-depressed and remitted adolescents. Additionally, it is intuitively obvious that recalling positive memories per se is a fun activity and should improve mood (Tamir, 2009).

Similarly, researchers have focused on retrospective cognitive reappraisal, that is, the case when individuals reframe negative past events as benign, valuable, or even beneficial (Garnefski, Kraaij, & Spinhoven, 2001; Rusting & DeHart, 2000; Wisco & Nolen-Hoeksema, 2010). For instance, a person who has recovered from COVID-19 might retrospectively view the disease as a call to help others make similar recoveries. Consequently, the modified memories of painful experiences become suitable for positive retrieval, and hence, may be involved in the mechanism of positive distraction.

## Is Mood-Repair Potency Limited to Positive Memories?

However, the literature review revealed an important gap. Taken together, both automatic and deliberate accounts for mnemonic promotion of positive affect suggest that when faced with undesirable emotional states, individuals cope by retrieving exclusively pleasant memories. To the best of our knowledge, there is no research in the field that would disprove the assertion that the method of mnemonic mood repair is limited to positive memories.

The present study departed from the previous literature in the way we hypothesised that unhappy autobiographical memories serve the goal of emotion regulation without being transformed into a positive mode.

For further reasoning, it is important to reckon social comparison as a potential source of emotion regulation (Augustine & Hemenover, 2009). It is assumed that a person could feel better after performing downward comparisons with those doing worse than themselves (Aspinwall & Taylor, 1993). However, downward social comparisons with real persons have the potential to evoke guilt in the comparer because they violate personal morality and compete with empathy (White, Langer, Yariv, & Welch, 2006).

We speculated that unhappy autobiographical memories could boost the mood, not by positive refocusing from the current concern to some irrelevant pleasant thoughts but by a specific form of cognitive reappraisal, that is, the positive reappraisal of the mismatch between the past and the present. Considering the concept of downward social comparisons, it is termed here as ‘retrospective downward autobiographical comparison’. While in the literature on emotion regulation, reappraisal is routinely thought as rethinking of an emotional event in the past, the ‘retrospective downward autobiographical comparison’ proposed in the current study involves a reappraisal of one’s current circumstances in the contrast to past emotional events. It may be stated that the former type of reappraisal is a past-targeted process that serves to modify existing memories, whereas the latter type of reappraisal is a present-targeted process that revises the perception of the present by collating with the past.

When such comparison reflects favourably on the current circumstances, one might feel that ‘things have changed for the better’ and consequently, experience a hedonic shift in the affect. Our method of reasoning partially corresponds with a distinct form of retrospective cognitive change denoted as ‘putting into perspective’ by Garnefski et al. (2001). It refers to noticing relativity or even paltriness of current negative experiences when compared to other life events. Although the authors emphasised the importance of such a comparison, they interpreted it in terms of general probability, for example, one could think that it all could have been much worse; one could tell oneself that there are worse things in life, etc. We speculated that addressing a concrete moment in the personal past may lead to more pronounced affective consequences.

Various cases illustrate this point. For instance, imagine a student whose sad mood is induced by a moderate failure in the academic domain. If she retrieves a positive memory from the relevant domain (e.g. passing exams successfully), it may make her feel even worse (here and now loses when compared with there and then). In contrast, if she retrieves an associated negative memory (e.g. threatened to be expelled from the university), her appraisal of the current situation would benefit from this comparison. Another example may be taken from the romantic relationship domain. Consider a woman experiencing melancholy due to her partner’s inadvertence. If sweet memories about her tender first love will emerge in her mind in this situation, the contrast between the good past and disappointing present may increase her dissatisfaction. Conversely, the sad memories of loneliness in the past may help her look at current relationships through rose-tinted glasses.

## Method

The present study was designed to empirically examine the influence of deliberately retrieved negative autobiographical memories on the emotional state of non-depressed young adults. The main hypothesis posits that the feeling that things have changed for the better may arise in response to recalling a negative memory employing the mechanism of ‘retrospective downward autobiographical comparison’, that is, a comparison between the worse past and better present. In other words, after a deliberate experience of negative autobiographical memories, we tested the prediction that participants would enjoy the present moment more than before.

Notably, the present study focused on the uncontemplated effect of intentional negative recall. Taking into account, people’s tendency to retrieve positive memories as a controlled mood regulation strategy (Ortner, Briner, & Marjanovic, 2017; Rusting & DeHart, 2000) and expect negative memories to induce negative mood (Hernandez, Vander Wal, & Spring, 2003; Öner & Gülgöz, 2018; Pond & Peterson, 2020), we sought to avoid the influence of such bias on the emotional outcomes of the recall. Therefore, this study dealt with implicit emotion regulation which has been shown to combine habitual strategies with continual adjustment to the contingencies. We predict that intentional, but purposeless, negative recall may support all three consecutive stages of implicit emotion regulation, as asserted by Koole, Webb, and Sheeran (2015). First, it may activate emotion regulation goals. Second, it may serve as a situational affordance for selecting appropriate emotion regulation strategies. Third, it may maintain the process of implementing the chosen strategy until the goal is achieved. Therefore, participants did not receive explicit incentives to change their existing moods.

In the present study, we also examined our speculation on the potential positive affective consequences of negative content, accommodating some insights from the field of media psychology. In an attempt to explore why people enjoy watching sad movies and reading sad fictional literature even if there is no redeeming happy end, the authors proposed that they may tolerate negative affective states temporarily for the sake of better emotional outcomes in the future (Mar, Oatley, Djikic, & Mullin, 2011). The mechanism of restoring a negative mood by comparing oneself with a fictional character depicted as more unfortunate following the insight that one’s concerns are not the worst has been identified among various motivations behind the puzzling popularity of tragic plots (Koopman, 2015). Consistent with this view and conceptually related to downward social comparisons, previous literature has shown that individuals with specific problems (e.g. loneliness) select and retrospectively highly appreciate films that include a pessimistic portrayal of people with similar problems (Mares & Cantor, 1992). However, the emotional benefits of a downward comparison are absent when identifying with fictional characters. Strong identification leads to a higher probability of experiencing the same emotional state as experienced by the target (Mar, Oatley, Djikic, & Mullin, 2011). Moreover, taking a first-person (rather than a third-person) perspective enhances identification with a narrative protagonist (Samur, Tops, Slapšinskaitė, & Koole, 2021).

Correspondingly, the phenomena of vicarious memories, that is, memories representing events that happened to others, even fictional protagonists, have recently received substantial interest in the field of autobiographical memory (Pillemer, Steiner, Kuwabara, Thomsen, & Svob, 2015). It has been reported that the phenomenological and functional qualities of vicarious memories closely resemble those of autobiographical memories. However, vicarious memories are typically rated as less vivid, less personally important, and more negative than autobiographical memories (Pond & Peterson, 2020). There is a theoretical assumption that negative vicarious memories may serve a self-enhancement function through a downward social comparison and impact emotion regulation (Thomsen & Pillemer, 2017).

Considering the probable self-enhancement function of negative vicarious memories, further empirical research is needed to determine whether vicarious memories are identical to autobiographical memories in emotion regulation. Therefore, we introduced memories on behalf of a favourite fictional character as the control condition.

The following tentative hypotheses represent the expected findings consistent with the study aims:

**H1:** Intentional positive autobiographical recall in the absence of an explicit goal of emotion regulation will ameliorate the participants’ mood implicitly through the mechanism of positive distraction from the current concerns.
**H2:** Intentional negative autobiographical recall in the absence of an explicit goal of emotion regulation will ameliorate the participants’ mood implicitly through the mechanism of retrospective downward autobiographical comparison, that is, a comparison of the current concerns against their more negative memories.
**H3:** Positive recall on behalf of a favourite fictional character will produce a similar but weaker improvement of mood. The expected effect will arise due to the distractive potential of entering a fictional world enhanced by identification with a protagonist.
**H4:** Negative recall on behalf of a favourite fictional character will have a reverse effect on implicit emotion regulation in comparison with negative autobiographical recall. This expected result will be due to the use of first-person narration, which evokes identification with the protagonist and, consequently, inhibits the downward social comparison emotion regulation strategy.

### Participants

Ninety-nine psychology students (*M*_age_ = 20.31, *SD* = 2.06; 68 females) participated in the study for partial course credit. The sample size was calculated a priori using the ANOVA: repeated measures, within factors option in G*Power 3 (Faul, Erdfelder, Lang, & Buchner, 2007) specifying a medium effect size $\eta_{p}^{2}$ = .25) with a power of 1 – β  =  .90. Considering that two factors were included into design, the recommended minimum sample of 23 was multiplied by four in accordance with the claim made by Brysbaert (2019). Consequently, the required sample size was determined to be 92. Therefore, the present study was appropriately powered.

The participants had no history of treatment for any psychiatric conditions. They signed an informed consent form approved by the Ethics Committee of the Faculty of Psychology of Lomonosov Moscow State University under the Declaration of Helsinki (Project No. 2019/64).

### Measures

#### Emotional State

Several competing approaches exist for assessing mood and emotion (see for review, Gray & Watson; 2007). In this study, we chose the outcome measures based on Watson and Tellegen’s hierarchical model of affect structure, which proposes two high-order largely unipolar dimensions of affective space: positive affect (PA) and negative affect (NA). Since the self-report methodology is prone to various biases, particularly, to cultural stereotypes about the nature of emotional experience, their self-report Positive and Negative Affect Schedule (PANAS, Watson, Clark, & Tellegen, 1988) was not used. Similarly, we speculated that the folk belief that negative memories ruin positive mood might disguise their regulative power in the relevant experimental condition if emotions are measured explicitly (Ford & Gross, 2018). Departing from the self-report methodology, we assessed emotional states using the indirect projective method: The Implicit Positive and Negative Affect Test (IPANAT, Quirin, Kazén, & Kuhl, 2009). However, the authors do not equate the term ‘implicit affect’ with the ‘unconscious’. Consistent with the proposition that emotions are, per definition, the states of consciousness and not necessarily reflective (Lieberman, 2019), they posit that the IPANAT allows the assessment of automatic and pre-reflective components of the affective experience. Several recent studies have used the IPANAT, demonstrating the compatibility of the results of this novel method compared to the traditional self-report methods. For instance, in Quirin, Bode, and Kuhl (2011) study, participants performed the IPANAT and self-report mood adjective checklist before and after exposure to a fear-inducing video. As expected, both implicit and explicit PA decreased immediately after viewing a threat-related video and increased after a time delay, detecting the recovery of a neutral emotional state. However, the IPANAT is more sensitive to emotion dynamics and the corresponding behavioural variables. Concurrently, examining whether a mindfulness exercise improves explicit and implicit negative moods, Remmers, Topolinski, and Koole (2016) revealed the same pattern of results across both measures.

The IPANAT procedure requires participants to rate the degree to which nonsense words (e.g. SAFME, VIKES, TUNBA, PEVIL, BELNI, and MADDO) convey various feelings. To conceal the real target of the IPANAT, the participants are encouraged to guess the meaning of the nonsense words to examine their linguistic intuition.

Since the list of stimuli was proven to be emotionally neutral across 10 countries, including Russia (Quirin et al., 2018), it is assumed that, in accordance with the affect infusion principle, participants project their current affects on this material. Participants rate six nonsense words with respect to three positive and three negative emotional states using a 4-point Likert scale ranging from ‘doesn’t fit at all’ to ‘fits very well’. According to the authors, the IPANAT reveals two orthogonal dimensions: positive affect (PA) and negative affect (NA). Averaging adjective scores derived from positively and negatively valenced adjectives present the scores for PA and NA, respectively.

The Russian version of the IPANAT is a valid and reliable instrument with satisfactory psychometric properties (Mitina, Padun, & Zelyanina, 2017). It is noteworthy that 64 nonsense words are available to construct non-recurring lists for repeated measures.

#### Memory Tasks

In the autobiographical memory task, participants were asked to give brief but detailed descriptions of the event they experienced more than a year ago that lasted over minutes or hours but no longer than a day. In the ‘happy’ condition, they received the following instructions: ‘This memory should be a highly positive experience that made you feel happy, satisfied, and lucky at the time it occurred’. In the ‘unhappy’ condition, they received the opposite instructions: ‘This memory should be a highly negative experience that made you feel unhappy, disappointed, and helpless at the time it occurred’.

In the vicarious memory task, participants were asked to do the same on behalf of their favourite fictional characters. We encouraged participants to put themselves in their favourite character’s shoes and tell the stories as if they were them. The exact instruction was: ‘Imagine yourself to be your favourite fictional character and then recollect an event from her/his perspective, as if you were they’.

#### Time Perspective

To control for individual differences in personal attitudes toward their experience, we included the Zimbardo Time Perspective Index (ZTPI, Zimbardo & Boyd, 1999) subscales tapping the two dimensions: Past Positive (PP, 9 items) and Past Negative (PN, 10 items).

#### Empathy for a Fictional Character

To control for individual differences in becoming absorbed in the feelings and actions of the fictional characters, we included the fantasy subscale from the Interpersonal Reactivity Index (IRI, Davis, Luce, & Kraus, 1994) consisting of seven statements.

For both scales, participants were asked to indicate the extent to which each of the statements was true for them on a 5-point Likert scale.

#### Thematic Analysis

Considering the emphasis on the possible contribution of negative memories in mood regulation, the contents of negative memories were analysed according to the themes that emerged from the narratives. Three coders independently read the memory descriptions to develop a list of content categories. Then, the coders inspected the memory descriptions again and assigned them to the most relevant categories that emerged from the previous coding procedure. The final attribution was based on the majority’s decision.

### Procedure

Participants attended four sessions held approximately one week apart. Upon their first arrival at the laboratory, participants were informed that the study dealt with various kinds of memories and their linguistic intuition. They then signed a consent form and completed the IRI and ZTPI subscales. Each session started with an indirect assessment of the emotional state by completing the IPANAT. Then, the participants performed a memory task randomised across sessions and participants. Immediately after performing the memory task, the participants completed the IPANAT again. Eight non-recurring lists of the IPANAT stimuli were used; therefore, at each assessment, the participants dealt with a novel set of nonsense words. Three research assistants communicated with each participant individually. The questionnaire was presented online on the Google platform; however, the participants received the link only in the laboratory. Each session, except for the first session, took approximately 20 minutes. The participants were debriefed after the last session, where they learned about the true purpose of the study. None of the participants reported that they realised the true goal of the study before being debriefed.

## Results

To exclude the possibility that condition-specific effects on emotion regulation were due to baseline differences in moods prior to manipulation, pre-test IPANAT scores were compared between the four conditions. There were no significant differences in PA, *F*(3, 98) = 0.664, *p* = .575, nor for NA, *F*(3, 98) = 1.655, *p* = .177. Both the PA and NA scales showed adequate internal consistency, with Cronbach’s alpha of .83 for each.

Table 1 presents the means and standard deviations of the IPANAT scores at pre- and post-tests for all memory tasks (autobiographical positive, autobiographical negative, vicarious positive, and vicarious negative).

**Table 1:** Descriptive Statistics for Means and Standard Deviations of the IPANAT Score

	Autobiographical Memory Task								Vicarious Memory Task							
	Positive Memory				Negative Memory				Positive Memory				Negative Memory			
Statistic	PA		NA		PA		NA		PA		NA		PA		NA	
	pre	post	pre	post	pre	post	pre	post	pre	post	pre	post	pre	post	pre	post
*M*	2.06	2.09	2.08	1.98	2.07	2.49	2.09	1.76	2.12	2.22	2.02	1.94	2.11	1.98	2.04	2.1
*SD*	.42	.52	.41	.52	.53	.52	.54	.48	.51	.50	.44	.43	.51	.51	.45	.50

*Note*. *N* = 99; PA = positive affect; NA = negative affect; pre = before performing the memory task; post = after performing the memory task.

Two repeated-measures mixed ANOVAs were conducted with PA and NA as dependent variables separately with time (before vs. after the memory task) and two within-subject factors of memory valence (positive vs. negative) and memory attribution (autobiographical vs. vicarious on behalf of one’s favourite fictional character).

The ANOVA on PA revealed a main effect of time, *F*(1, 98) = 25.71, *p*  < .001, $\eta_{p}^{2}$ = .208. On average, participants scored higher on positive affect after performing the memory tasks. There was a main effect of attribution of the retrieved memory to one’s past or a favourite fictional character, *F*(1, 98) = 4.66, *p *= .03, $\eta_{p}^{2}$ = .045. The PA associated with autobiographical memories appeared to be slightly higher than those associated with alter-biographical memories. Importantly, the analysis also indicated reliable interactions: time × attribution, *F*(1, 98) = 25.53, *p *< .001, $\eta_{p}^{2}$ = .207, valence x attribution, *F*(1, 98) = 48.24, *p *< .001, $\eta_{p}^{2}$ = .330, and time × valence × attribution, *F*(1, 98) = 56.61, *p *< .001, $\eta_{p}^{2}$ =  .366.

Follow-up analyses revealed that performing positive memory tasks inflated the PA when the participants retrieved vicarious memories, *F*(1, 98) = 6.95, *p *= .01, $\eta_{p}^{2}$ =  .066 but not after autobiographical retrieval, *F*(1, 98) = 0.548, *p *= .461. Participants’ performance on negative memory tasks led to a mild decrease in PA in the vicarious condition, *F*(1, 98) = 10.28, *p *= .002, $\eta_{p}^{2}$ =  .095, whereas PA increased extensively in the autobiographical condition, *F*(1,98) = 88.91, *p *< .001, $\eta_{p}^{2}$ = .456. Thus, a negative recall produced reverse effects on the PA depending on the examination of one’s experience or the biography of a fictional character.

A congruent pattern of data was obtained for NA scores. The ANOVA on the NA indicated a main effect of time, *F*(1, 98) = 28.63, *p *< .001, $\eta_{p}^{2}$ = .226. On average, participants scored lower on negative affect after performing the memory tasks. The analysis detected significant crossover interactions: time × attribution, *F*(1, 98) = 26.56, *p *< .001, $\eta_{p}^{2}$ = .213, attribution x valence, *F*(1, 98) = 37.16, *p *< .001, $\eta_{p}^{2}$ = .275, and time x valence x attribution, *F*(1, 98) = 24.78, *p *< .001, $\eta_{p}^{2}$ = .202.

A follow-up inspection demonstrated an identical slight decline in NA in both the autobiographical and vicarious conditions after performing positive memory tasks, *F*(1, 98) = 6.60, *p *= .01, $\eta_{p}^{2}$ = .059 and, *F*(1, 98) = 4.28, *p *= .04, $\eta_{p}^{2}$ = .042, respectively. In contrast, performing negative memory tasks inhibited the NA dramatically in the autobiographical condition, *F*(1, 98) = 52.11, *p *< .001, $\eta_{p}^{2}$ = .330, whereas the same task in the vicarious condition did not evoke any effect on the NA, *F*(1, 98) = 2.55, *p* = .113.

To identify the role of additional self-reported measures, we calculated correlations between the ZTPI and IRI scores and the IPANAT difference scores (post-test minus pre-test scores) in the four conditions. Nevertheless, none of these measures were correlated with one exception: ZTPI negative past and the NA difference for positive autobiographical recall (*r* = .344, *p* < .001). Considering that NA typically decreases in this condition, it may be assumed that an aversive view of the personal past interferes with the mitigation of negative affect with positive memories. This assumption was supported by the fact that elucidating the ANOVA with the ZTPI negative past scores as a covariate substantially boosted the statistical power, *F*(1, 98) =  8.66, *p *< .001, $\eta_{p}^{2}$ =  .151.

**Figure 1:**
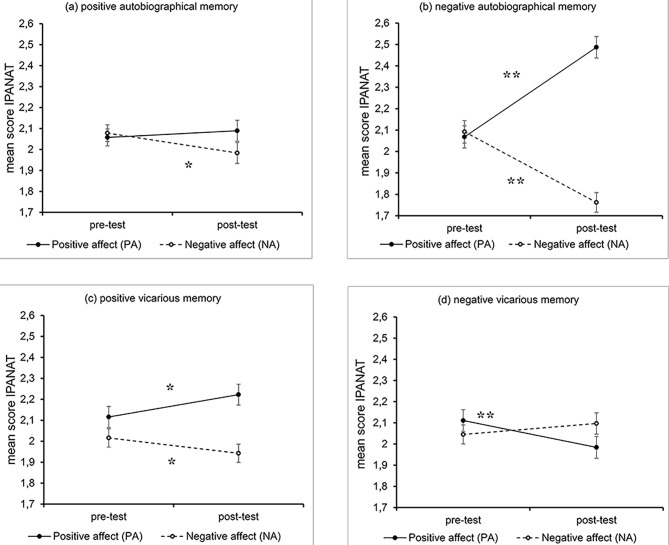
Mean IPANAT Scores for Positive Affect (PA) and Negative Affect (NA) Before and After Performing Memory Tasks *Note.* Error bars indicate standard errors of the mean. A significant result of the simple effects test, indicating a difference between pre- and post-test scores, is marked with a star. *p < .05. **p < .01.

Figure 1 displays the mean scores of the positive and negative affect on the IPANAT as a function of memory tasks.

### The Content of Negative Memories

To elucidate the results further, we examined whether the contents of negative memories produced by the participants somehow influenced the following emotional states. Although the categories were not pre-decided, the analysis resulted in categorizing all the memories into eight corresponding themes.

The following categories were identified: 1) Death of relative/friend/pet (e.g. ‘The two memories immediately came to my mind. The death of my grandmother and the death of my lovely cat. These events have merged somewhat because I experienced similar emotions. These two creatures, pardon the expression, were the closest to me. In the first case, I could do nothing since I was too young. But for the second case, I still blame myself. She (the cat) was sick and jumped out the window to catch a sparrow.’); 2) Separation/End of relationships/Loneliness (e.g. ‘The last conversation with my boyfriend, when he was back from the army. I remember us staying near the army barrack. It was windy, cold, and rainy. He told me not to contact him anymore, not to expect anything from our relationships.’); 3) Humiliation (e.g. ‘I was catastrophically humiliated by my classmate. She took picture of me at the gym changing room by stealth. And then she sent the photo to my boyfriend by noticing the imperfections of my figure. I could not stop crying when I found out about it.’); 4) Incompetence in the academic domain (e.g. ‘Last year I was told that I had to present my project a week earlier than it was scheduled. I tried to cope with the time pressure, but I was very worried, felt overworked, and helpless. I felt that it would be very difficult to handle and that I didn't have the strength to do it.’); 5) Own/Relative’s disease (e.g. ‘The day Dad told me that Mom had been diagnosed with cancer.’); 6) Conflict/Betrayal (e.g. ‘First escape from home after a terrible quarrel with my mother. I spent all night at a staircase asking myself, “Why is life being so unfair to me?” I felt everything went wrong. I felt so unlucky, so stupid, so miserable.’); 7) Parental conflict/divorce (e.g. ‘I was eight when my parent’s marriage was broken. Of course, I could not figure out why my Dad was not at home. One day, my grandmother told me that they no longer lived together. I was really shocked. I could not make heads or tails of it.’).

Since the positive effect of negative recall was possible due to reappraisal in its traditional form, we carefully examined the verbal marks indicating such previous reappraisal and introduced the eighth category ‘Reappraised events’. The following record may exemplify this category: ‘There are moments in the past that bring a shadow of regret, but the understanding immediately comes that these situations served as a powerful stimulus for me, they shaped my character and without them, many great events would not have happened in my life.’

The modal category for the participants was ‘Incompetence in the academic domain’ (24.2%). The second most frequently observed category was ‘Separation/End of relationships/Loneliness’ (20.2%). The third and fourth common categories (16.2%) were ‘Death of relative/friend/pet’ and ‘Conflict/Betrayal’. Contrary to these concerns, the category ‘Reappraised events’ was mentioned among the rarest (6.1%) and, therefore, did not significantly influence the main results.

Considering the possible correspondence between the susceptibility to the positive effect of negative autobiographical recall and the content of retrieved memories, we split the sample into two unequal groups. The ‘boosters’ group involved those participants whose PA and NA synchronically increased and decreased, respectively, after performing negative autobiographical memory tasks (*N* = 74). The ‘non-boosters’ group involved those participants whose PA or NA did not follow the pattern described above (*N* = 25) (see Table 2). The analysis revealed that both subgroups reported similar frequencies of memories referring to each of the eight categories; χ^2^ (7, 99) = 1.763, *p* = .972.

**Table 2:** Percentages and Frequencies of Negative Memories

	Boosters (*N* = 74)		Non-Boosters (*N* = 25)	
Content Category	Frequency	Percent	Frequency	Percent
Death of relative/friend	12	16.25	4	16.00
Separation/End of relationships/Loneliness	15	20.30	5	20.00
Humiliation	7	9.50	1	4.00
Incompetence in the academic domain	17	23.00	7	28.00
Own/Relative’s disease	5	6.80	1	4.00
Conflict/Betrayal	11	14.90	5	20.00
Parental conflict/ divorce	2	2.70	1	4.00
Reappraised events	5	6.80	1	4.00

*Note.* Sorted by content category and susceptibility to the positive effect of the negative recall subgroup. The frequencies and percentages of negative autobiographical memories classified into 8 content categories described above for the two subsamples—‘boosters’ and ‘non-boosters’.

## Discussion

Although remembering may be an effective tool for emotion management for several reasons (Pasupathi, 2003; Pillemer, 2009), there is a dearth of research on the diversity of mechanisms that engage with the function of remembering to enhance positive and diminish negative moods.

People’s negative personal memories, when functional, have been shown to serve predominantly directive functions by taking lessons and applying those lessons to the current problems (Rasmussen & Berntsen, 2009). Accordingly, some ‘emotional lessons’ may be extracted from negative memories to resolve current concerns. To the best of our knowledge, no prior studies have examined the possibility that memories of negative past events per se could help people to view the current situation in more benign terms and, consequently, regulate one’s mood. The present study focused on the idea that the utility of negative memories for mood enhancement may imply a specific kind of retrospective cognitive reappraisal. In contrast to rethinking past negative events differently (retrospective reappraisal), the retrospective downward autobiographical comparison proposed here induces a positive mismatch between past negative experiences and the current situation, that is, the feeling that ‘since then, things have changed for the better’. The discrepancy between an aversive memory and an acceptable present seems to be a probable mechanism for positive mood induction.

The present study avoided directing the participants to goal-driven mood management. The rationale behind this was the assumption that metacognitive beliefs concerning how autobiographical memory works may contribute to individuals’ mnemonic behaviour (Wang et al., 2015). Since it seems highly likely that people believe in the negative influence of negative memories on their moods, it may oppose expected mood-repair effect after deliberate retrieval. To prevent such a scenario, the positive and negative components of mood were assessed using an indirect test (the IPANAT).

The results replicated and elucidated previous data on the mood-repair effect by deliberate recall of positive memories. Positive memory tasks significantly decreased the negative component of the mood (NA) in both autobiographical and vicarious conditions. Notably, an increase in positive affect (PA) was observed in the vicarious condition only, while it remained the same after the autobiographical recall. This asymmetry may be due to the entertaining nature of vicarious memory tasks. Putting oneself into one’s favourite fictional character’s shoes at the happiest moments of the story might be, in itself, a powerful mood regulation strategy.

The most striking finding from this study is the reverse effect of negative memory tasks in autobiographical and vicarious conditions. A decrease in PA and stasis of NA was detected in the vicarious condition. In contrast, negative autobiographical recall, consistent with our predictions, consistently boosted PA and inhibited NA. The former result may be attributed to identification with a chosen fictional character, which determines emotional mimicry. From our perspective, the latter result may support the idea that retrospective downward autobiographical comparison serves as a mechanism behind employing negative personal memories for emotion regulation.

It is noteworthy that the obtained result could not be attributed to the specific thematic content of memories. In this study, all negative autobiographical memories were grouped into a relatively small number of categories (*N* = 8). No significant differences were found in the frequency of memories assigned to each of the categories between the participants divided according to their mood shift after performing the memory tasks (boosters vs. non-boosters). In this study, the most widespread negative memories addressed separations and failures in the academic domain. These results are consistent with numerous data on the significance of self-mastery and communion themes in young adults’ autobiographical narratives, which, in turn, mirror the most powerful motives of this age cohort (see for details, McAdams, Hoffman, Day, & Mansfield, 1996).

At the outset of the study, it was expected that individuals who view their past through rose-tinted glasses would benefit more from positive autobiographical recall, whereas individuals with a negative bias toward their past would have difficulty resorting to an account of positive memories. Alternatively, such rose-tinted glasses could contradict the proposed mood-repair effect of negative memories, whereas a negative bias towards one’s past probably makes the effect more articulated. In fact, only high scores on the ZTPI negative past correlated with a decrease in NA after the positive memory task, highlighting this kind of negative bias as an obstacle to eliminating mood negativity.

### Conclusions

This experimental study is the first to examine whether negative autobiographical memories serve as a counterpart to positive ones in emotion regulation. This possibility was proposed when considering that negative memories may evoke a feeling that things are going better now as compared to the past. Consequently, this positive discrepancy may improve one’s affective state. The proposed hypothesis was tested in 99 healthy, young adults. In general, the claim that performing positive memory retrieval is a sufficient strategy for mood management was replicated. More importantly, the current study extended prior research by detecting the moderate but consistent effect for the PA and NA after a negative autobiographical memory task, in a way that participants increased their PA and decreased their NA at post-test. Notably, the effect took place spontaneously without the explicit goal of changing the existing mood and addressed implicit emotion regulation. These results go beyond previous reports, showing that a retrospective downward autobiographical comparison approach may provide useful insights into the functionality of autobiographical memory.

### Limitations and Future Directions

Undoubtedly, much remains to be investigated before we have a clear picture of all the conditions under which negative memories may reduce mood negativity and promote mood positivity. Further research should examine the nuances of candidate memories with respect to their content, age, emotional intensity, and the level of discrepancy between the recalled event and the current concern. We are also aware that employing negative memories for emotion regulation functions can exhibit cultural differences. For instance, since it has been shown that people with East Asian cultural backgrounds are able to tolerate negative memories successfully (Nourkova, 2020; Spencer-Rodgers, Peng, & Wang, 2010), they could benefit more from negative memories than people with European and North American cultural backgrounds. However, the current paper contributes to our understanding of emotion regulation and has demonstrated a possible and previously overlooked application of negative autobiographical memories in diminishing negative emotions or distress.
